# BODY WEIGHT INFLUENCES MUSCULOSKELETAL ADAPTATION TO LONG-TERM VOLUNTARY WHEEL RUNNING DURING AGING

**DOI:** 10.1093/geroni/igac059.1609

**Published:** 2022-12-20

**Authors:** Yukiko Kitase, Vallejo Julian, Yixia Xie, Mark Dallas, Sarah Dallas, Mark Johnson, Michael Wacker, Lynda Bonewald

**Affiliations:** Indiana University School of Medicine, Indianapolis, Indiana, United States; University of Missouri-Kansas City, School of Dentistry and Medicine, Kansas City, Missouri, United States; University of Missouri-Kansas City, School of Dentistry, Kansas City, Missouri, United States; University of Missouri-Kansas City, School of Dentistry, Kansas City, Missouri, United States; University of Missouri-Kansas City, School of Dentistry, Kansas City, Missouri, United States; University of Missouri-Kansas City, School of Dentistry, Kansas City, Missouri, United States; University of Missouri-Kansas City, School of Medicine, Kansas City, Missouri, United States; Indiana University School of Medicine, Indianapolis, Indiana, United States

## Abstract

Frailty is a key hallmark of aging and exercise has been shown to delay aging effects. This study was initiated based on the hypothesis that voluntary wheel running (VWR) starting at 12 mo until 18 or 22 mo of age would benefit the female murine musculoskeletal system. Based on the final body weight, the mice were separated into high (HBW) and low body weight (LBW) subgroups. Beneficial effects of VWR were observed on soleus muscle mass and contractile force at both ages, although HBW led to greater increases at 22 mo. VWR increased fiber cross-sectional area by 20%, leading to more type I and fewer IIA fibers in soleus. HBW mice were resistant to age-related decline in Extensor digitorum longus (EDL) mass and contractile force. EDL in 18 mo HBW also showed 15% higher contractile force following VWR while muscle from 18 & 22 mo LBW responded to VWR with greater osteocyte protective factor secretion. Skeletal adaptation to VWR was also dependent on body weight, with HBW showing higher femoral cortical thickness and area under sedentary conditions. VWR maintained osteocyte dendrite number in HBW. VWR increased periosteal and endosteal circumferences in HBW, suggesting compensation for loss of material strength. Consistent with this, VWR maintained higher bone mechanical properties in 18mo LBW. In summary, VWR alters musculoskeletal parameters depending on body weight with HBW contributing to more muscle mass and strength to prevent sarcopenia while bone retains better mechanical properties in LBW but HBW contributes structural modification to prevent osteopenia.

